# Canonical Single Nucleotide Polymorphisms (SNPs) for High-Resolution Subtyping of Shiga-Toxin Producing *Escherichia coli* (STEC) O157:H7

**DOI:** 10.1371/journal.pone.0131967

**Published:** 2015-07-01

**Authors:** Sean M. Griffing, Duncan R. MacCannell, Amber J. Schmidtke, Molly M. Freeman, Eija Hyytiä-Trees, Peter Gerner-Smidt, Efrain M. Ribot, James L. Bono

**Affiliations:** 1 PulseNet Next Generation Subtyping Methods Unit, Division of Foodborne, Waterborne and Environmental Diseases, Enteric Diseases Laboratory Branch, Centers for Disease Control and Prevention, Atlanta, Georgia, United States of America; 2 United States Meat Animal Research Center, United States Department of Agriculture, Agricultural Research Service, Clay Center, Nevada, United States of America; University of Münster, GERMANY

## Abstract

The objective of this study was to develop a canonical, parsimoniously-informative SNP panel for subtyping Shiga-toxin producing *Escherichia coli* (STEC) O157:H7 that would be consistent with epidemiological, PFGE, and MLVA clustering of human specimens. Our group had previously identified 906 putative discriminatory SNPs, which were pared down to 391 SNPs based on their prevalence in a test set. The 391 SNPs were screened using a high-throughput form of TaqMan PCR against a set of clinical isolates that represent the most diverse collection of O157:H7 isolates from outbreaks and sporadic cases examined to date. Another 30 SNPs identified by others were also screened using the same method. Two additional targets were tested using standard TaqMan PCR endpoint analysis. These 423 SNPs were reduced to a 32 SNP panel with the almost the same discriminatory value. While the panel partitioned our diverse set of isolates in a manner that was consistent with epidemiological data and PFGE and MLVA phylogenies, it resulted in fewer subtypes than either existing method and insufficient epidemiological resolution in 10 of 47 clusters. Therefore, another round of SNP discovery was undertaken using comparative genomic resequencing of pooled DNA from the 10 clusters with insufficient resolution. This process identified 4,040 potential SNPs and suggested one of the ten clusters was incorrectly grouped. After its removal, there were 2,878 SNPs, of which only 63 were previously identified and 438 occurred across multiple clusters. Among highly clonal bacteria like STEC O157:H7, linkage disequilibrium greatly limits the number of parsimoniously informative SNPs. Therefore, it is perhaps unsurprising that our panel accounted for the potential discriminatory value of numerous other SNPs reported in the literature. We concluded published O157:H7 SNPs are insufficient for effective epidemiological subtyping. However, the 438 multi-cluster SNPs we identified may provide the additional information required.

## Introduction

Shiga-toxin producing *Escherichia coli* (STEC) frequently causes infections from foodborne sources such as meat and raw produce [1]. Symptoms of STEC infection range from mild gastroenteritis and bloody diarrhoea to more severe outcomes, including sepsis and haemolytic uremic syndrome (HUS), with significantly higher morbidity and mortality among paediatric populations [2, 3]. In North America, STEC O157:H7 is the most common STEC serotype isolated from clinical samples and may account for more than 75,000 cases of foodborne illness and 15 to 20 outbreaks each year [3]. STEC O157:H7 is a clonal pathogenic bacterium with reservoirs in ruminants. It is transmitted in humans through contaminated food or water, contact with animals or by person-to-person contact. Subtyping *E*. *coli* O157:H7 collected from patients, ruminants, food, and field samples was recognized as valuable after a major outbreak at a restaurant chain in the western U. S. in 1993 [4].

Pulsed-field gel electrophoresis (PFGE) and multi-locus variable number tandem repeat analysis (MLVA) are used to subtype STEC O157:H7 by PulseNet, the national molecular subtyping network for foodborne disease surveillance. PFGE is considered the “gold standard” and MLVA provides further strain discrimination [5–8]. PFGE is a robust method that depends on variation in the size of restriction fragments. Pattern differences are believed to be largely a function of phage and indel genomic changes and do not always provide enough diversity for accurate subtyping among highly clonal organisms such as STEC O157:H7 [9–11]. As a consequence, PFGE is sometimes incapable of resolving epidemiologically confirmed, outbreak-associated isolates from a background of sporadic cases. MLVA often provides further resolution by targeting rapidly evolving short repeat sequences through serotype-specific protocols. While MLVA is a valuable supplementary test for subtyping a limited number of foodborne organisms, it is not a feasible replacement for PFGE due to its serotype-specificity.

SNP analysis has been successfully applied to the investigation of highly-clonal bacterial outbreaks, studies of bacterial molecular evolution, and the characterization or subtyping of routine clinical isolates [12–15]. Therefore, A SNP-based assay for STEC O157:H7 might provide additional benefits including improved tracking of phylogenetic relationships and faster results than PFGE or MLVA. Recently, multiple studies have recommended that SNPs could be used to subtype STEC O157:H7 isolates [13, 16–19]. For this reason, PulseNet has already begun to focus on the potential value of single nucleotide polymorphism (SNP)-based canonical subtyping panels [20, 21].

The goal of this project was to identify and validate a panel of discriminatory SNPs for high-resolution molecular subtyping of STEC O157:H7 strains. For such a panel to be useful for public health purposes, it needs to provide equivalent or better discriminatory power than the established PFGE or MLVA methods, as well as good agreement with epidemiological data. We report here on our progress.

## Materials and Methods

### Identification of SNP targets

Prior to this project, our group identified 906 potential SNPs for use in differentiating STEC O157:H7 isolates [21]. We applied comparative genomic resequencing (Nimblegen, Madison WI) to 11 O157:H7 strains to identify SNPs that were potentially of discriminatory value. Nine of the strains represented the different lineages seen in a phylogeny of O157:H7 based on PFGE *Xba*I DNA restriction enzyme patterns. The remaining two were chosen because they were phenotypically atypical; typical O157:H7 are unable to ferment sorbitol (Sorb-) or exhibit β-glucuronidase activity (GUD-), but these were Sorb+ and GUD+ and Sorb- and GUD+. We considered a SNP as potentially useful for high-resolution subtyping if multiple alleles were present among the majority of the 11 isolates. This process suggested 391 SNPs that we tested using the OpenArray platform (Life Technologies, Grand Island, NY) on our initial microarrays. Note that throughout the remainder of the text we will refer to microarrays designed to test our 391 SNPs as “initial” and the additional microarrays designed to test the 30 SNPs suggested by other authors as “follow up.”

While we were testing these 391 SNPs, other authors identified additional potentially useful SNPs. We selected 30 SNPs suggested by others for OpenArray testing on follow up microarrays, along with two previously examined SNPs to verify assay consistency (Table 1 and S2 Table) [13, 16–19]. A study of bovine and human strains, recommended thirteen potential SNPs. Four had already been examined in our initial microarrays, but we selected six for our follow up microarrays [19]. Another study suggested 32 SNPs which generated 39 SNP genotypes for 528 O157 strains and close relatives [13]. Nine were on our initial microarrays or examined in our initial survey of potential targets, leaving 23 remaining targets. We removed 12 that had a minor SNP prevalence below 5% and selected the remaining eleven. A study by Leopold *et al*. suggested 1,113 SNPs, which were organized by three successive evolutionary clusters [16]. These clusters are different from the 10 clusters we describe in this manuscript. We removed unclassifiable SNPs, SNPs that would likely have little impact on phylogeny, and those that were examined on our initial microarrays. This process left one target in Leopold’s cluster one, which we did not pursue because of space constraints and the assumption that cluster one SNPs would not provide critical phylogeny resolution. It also left twenty five in Leopold’s cluster three, three of which had been recommended by others with a minor allele frequency below 5% and we therefore removed from consideration [13]. This left 22 SNPs that we filtered by assigning tentative common haplotypes based on the data from eight of the eleven SNPs used to select our initial 391 SNPs. We selected three for follow up microarray testing. We also selected four of Leopold’s cluster two SNPs reported in a later paper (Table 1) [17]. Later, data suggested three targets from Clawson, *et al*., two targets from Leopold, *et al*., and one target from Manning, *et al*. might be redundant. We replaced these six targets with others from Bono, *et*. *al*. [19, 22].

**Table 1:** Summary of alternate targets on the follow up microarray.

SNP ID	SNP location	Cluster Disrupted?	Source	Comments
ECs0799	889133	Cluster 1	[19]	Reported in Humans
ECs3076	3015872	No	[19]	Reported in Humans
ECs4494	4538018	No	[19]	Reported in Humans
ECs1017	1124089	Cluster 1	[19]	Reported in Humans
ECs2000	1976735	Cluster 1	[19]	Reported in Humans
ECs5049	5137547	No	[19]	Reported in Humans
ECs2057	2041566	No	[16]	
ECs3881	3885057	No	[16]	
ECs4826	4891379	Cluster 1, Cluster 2	[16]	
	3798	No	[17]	
	5234150	No	[17]	
	1894260	No	[17]	
	2422218	No	[17]	
ECs4889	4964826	Cluster 1	[13]	
ECs0712	789194	No	[13]	
ECs5273	5398532	No	[13]	
ECs3609	3599366	No	[13]	
ECs3743	3744736	No	[13]	
ECs0721	797116	Included in discriminatory set; Cluster 1	[13]	
ECs2775	2717449	Cluster 3, 7, 9	[13]	
ECs3830	3838445	No	[13]	
ECs0942	1027219	Cluster 1	[13]	
ECs3942	3944571	Included in discriminatory set	[13]	
ECs1011	1115049	No	[13]	
	3293290	No	[16, 22]	Replacement
	105178	No	[22]	Replacement
	331010	Cluster 1	[22]	Replacement
	3836658	Cluster 1	[22]	Replacement
	4690438	No	[22]	Replacement
	1107987	No	[22]	Replacement
ECs0580	639975	No	[21]	Control
ECs3880	3884025	Cluster 1	[21]	Control

This table describes the 32 SNPs that were examined on the follow up microarrays based on SNPs recommended by other authors. Note that the “Disrupts tree?” column refers to the ten clusters that we describe later, rather than the clusters assigned by Leopold, *et al*. SNPs with the comment “Replacement” refer to SNPs that replaced those that were potentially redundant.

Aside from the follow up microarrays, two additional targets (B3193, 3,193,051 and B3133, 3,133,563) suggested by Bono, *et*. *al*. were tested using TaqMan PCR endpoint analysis on an Applied Biosystems 7500 Real-Time PCR System (Life Technologies).

### Screening of our 391 candidate SNPs and 30 additional targets

An initial panel of 128 of our 391 SNPs were pre-validated using hairpin primer PCR in collaboration with Biotrove Inc. (Woburn, MA) and used to test the OpenArray platform (now Life Technologies). The OpenArray platform is based on a small chip with 48 subarrays, each with 64 through-holes. Each hole contains a real time PCR reaction with a 33 nL reaction volume and two probes with different fluorescent dyes. A camera measures the light emitted from each hole and reports which probe fluoresces. The remainder of the 391 SNPs were tested on the initial microarrays as conventional TaqMan assays with FAM and HEX-labelled probes for the major and minor alleles designed using AlleleID 5.0 (Premier BioSoft, Palo Alto CA). All plates had one or more positive control isolates per chip.

While experiment layouts vary, a large number of samples can be tested simultaneously using this system. For the initial and follow up microarrays, we tested 121 historical sporadic and outbreak-associated isolates from the 1982–2006 PulseNet collection previously used to create a MLVA O157:H7 protocol, 46 isolates from 1996–2006 National Antimicrobial Resistance Monitoring System (NARMS) surveillance, and 2 fully sequenced control isolates (EDL933/Sakai). Our follow up microarray that tested the 30 additional SNPs also included eight additional controls to ensure that alternate SNPs were represented on the follow up microarrays (S3 Table). In total, we examined 177 O157:H7 STEC isolates. The primer and probe designs for these follow up microarrays were created in Primer Express 3.0.1 (Life Technologies) and VisualOMP (DNA Software, Ann Arbor, Michigan). Verification of selected discriminatory panel SNPs, data gaps, and two additional targets suggested by the USDA were examined using TaqMan PCR endpoint analysis on an Applied Biosystems 7500 Real-Time PCR System (Life Technologies).

### SNP analysis, selection, and quality control

The initial microarray results for the 391 SNPs were visualized and scored using the OpenArray SNP Genotyping program, version 1.02 (BioTrove Inc.) Later follow up microarrays were tested with OpenArray SNP Genotyping 1.4 (Life Technologies). SNPs that failed to produce consistent data on the initial or follow microarrays were dropped from consideration.

The final informative SNP panel was selected by a computer program called Haploview 4.2 and visual sorting [23]. Haploview was used to identify a SNP panel based on the analysis of 120 strains that were previously used to develop a MLVA protocol (S3 Table). Starting from stable simulation settings, Haploview selected different SNPs as discriminatory on different runs, as well anywhere between 19 and 34 SNPs. The SNP sets reported by each simulation were different even after we limited analysis to simulations that reported only 19 SNPs. However, each simulation consistently included a subset of ten stable discriminatory SNPs, which we accepted as part of our discriminatory panel.

We used these ten discriminatory panel SNPs to organize the 120 MLVA strains into a tree. We then visually searched through the remaining initial and follow up microarray data to identify additional SNPs that added epidemiologically useful reticulation to the tree. Additional SNPs were considered useful if they reticulated clusters that were not in agreement with MLVA, PFGE, and epidemiological data. Trees were created using FigTree v1.4.0 (http://tree.bio.ed.ac.uk/software/figtree/). We later expanded the tree to include 46 sporadic NARMS isolates.

### SNP panel subtyping comparison to MLVA and PFGE

We compared our final SNP discriminatory panel to MLVA and PFGE subtyping using 175 well-characterized, O157:H7 historical isolates (two isolates were removed due to missing MLVA or PFGE data). Genotype character data were imported directly into BioNumerics 5.10 (Applied Maths, Sint-Martens-Latem, Belgium) for analysis against known PFGE and MLVA clusters. Differences in Simpson’s index of diversity (D) were used to determine and compare discriminatory ability. Correlation between typing methods was assessed using the adjusted Wallace measure [24–26]. The number of groups generated by each method was also recorded.

### Search for additional discriminatory SNPs

After comparing our panel to MLVA and PFGE, we looked for additional useful discriminatory SNPs in ten clusters that did not agree with MLVA, PFGE, or epidemiological data. We did so by pooling DNA from each cluster to make sequencing libraries on an Illumina MiSeq Personal Sequencer following the TruSeq DNA Sample Preparation protocol (Illumina, Inc., San Diego, CA). To avoid generating SNPs with low informative value, only one representative isolate from each outbreak was included (S4 Table). DNA pools were generated by adding equal amount of DNA from each isolate to a final concentration of 30 ng/μl. A total of 29,773,598 Illumina reads were obtained for the 10 pools. Each pool contained 2,977,359 reads (stdev = 228,067 reads). Reads were trimmed in Geneious (Biomatters Limited, Auckland, New Zealand) for Illumina primers allowing one base mismatch with quality settings of 0.05 error probability limit and zero maximum ambiguities. After trimming, a total of 25,019,898 reads (average reads/pool = 2,501,989; standard deviation = 204,154) were mapped to the Sakai genome using the Geneious mapper with the Medium-low Sensitivity/Fast option with 5 iterations. SNPs were called that had a minimum coverage of 10 and a minor allele frequency above 0.05. Repetitive and prophage regions were removed from analysis. Each cluster reported a unique set of SNPs. We compared these sets of cluster SNPs to each other to identify SNPs that were shared among clusters and therefore potentially of greater discriminatory value.

## Results

**Fig 1:**
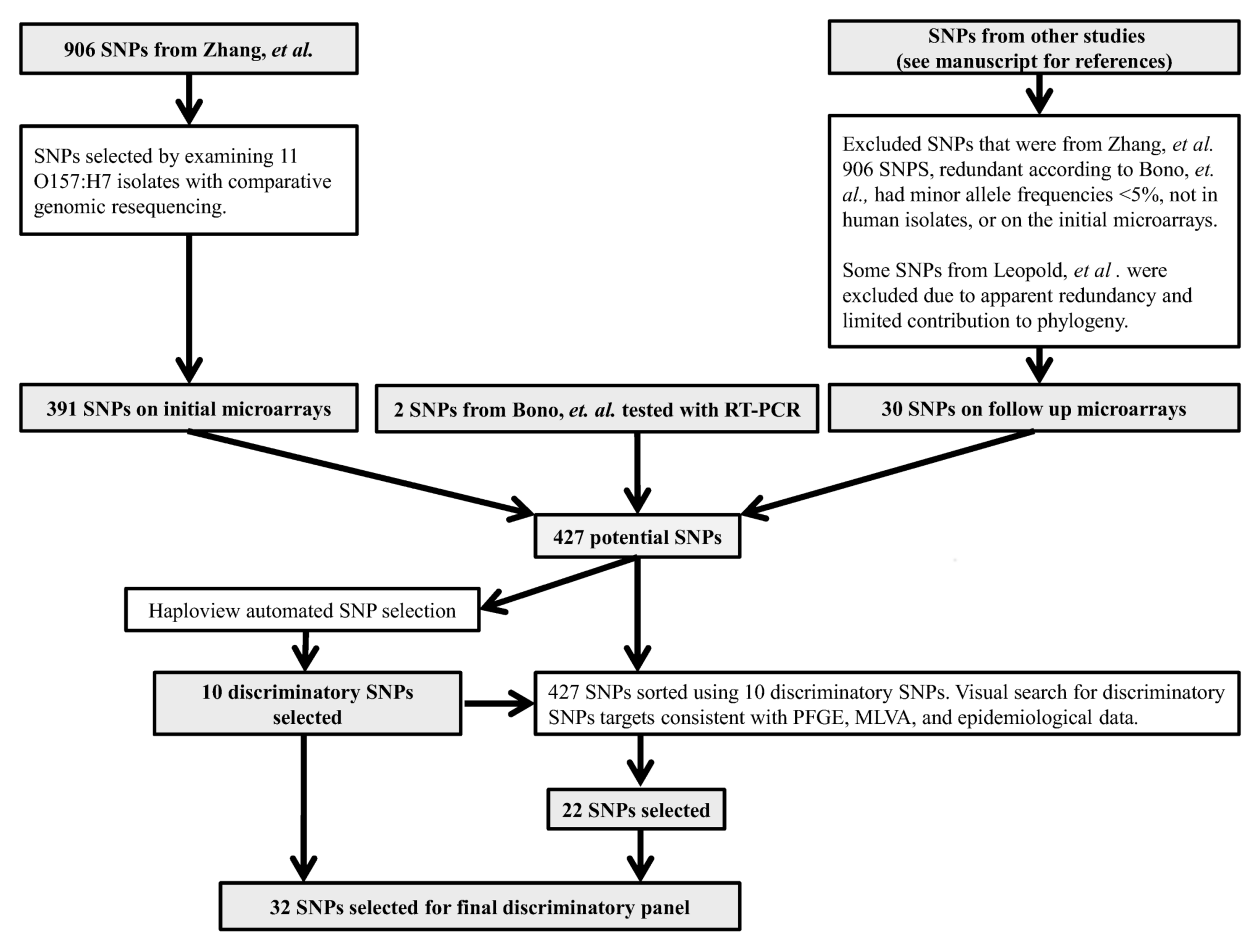
Workflow for final discriminatory SNP panel.

We limited our initial SNP panel selection, as well as pool verification and discovery, to an established, comprehensive set of STEC O157:H7 strains that were previously used to develop the serotype-specific MLVA subtyping method for PulseNet [20]. We found that 28 SNPs described the overall informative diversity found in our initial data for the 391 SNPs and therefore discarded the other 363 SNPs. Four additional targets suggested by others gave additional discriminatory value for epidemiological purposes according to our follow up microarray and standard RT-PCR data. This gave us a final panel of 32 SNPs (Table 2; Fig 1).

**Table 2:** Panel of 32 informative SNPs.

SNP ID	SNP Location	Major allele (frequency), minor allele (frequency)	Source
ECs0655*	731710	C (0.54), G (0.46)	[21]
ECS0333_70*	351109**	A (0.58), G (0.42)	[21]
ECs5290_1266	5419933	T (0.63), C (0.37)	[21]
ECs1360	1418991	A (0.64), G (0.36)	[21]
ECs5206	5307228	T (0.64), G (0.36)	[21]
ECs1414	1460599***	A (0.65), C (0.35)	[21]
ECs4293	4302784	G (0.65), T (0.35)	[21]
B3193	3193051	C (0.72), G (0.28)	[22]
ECs5052	5141169	A (0.81), G (0.19)	[21]
ECS2440_1200	2416801	A (0.82), G (0.18)	[21]
ECs2538	2512684	T (0.82), C (0.18)	[21]
ECs0418	445669	T (0.85), C (0.15)	[21]
ECs3540	3529080	C (0.85), T (0.15)	[21]
ECS0580_776	639975	G (0.86), T (0.14)	[21]
ECS2006_725	1984857	G (0.87), A (0.13)	[21]
ECs0721*	797116**	C (0.89), G (0.11)	[13]
ECs4387	4396804	T (0.89), G (0.11)	[21]
B3133*	3133563	G (0.9), A (0.1)	[22]
ECS3567_101*	3557744	G (0.9), A (0.1)	[21]
ECs4696_1167*	4732878	T (0.9), C (0.1)	[21]
ECs3942	3944571**	A (0.92), C (0.08)	[21]
ECs2915*	2864758	A (0.93), G (0.07)	[21]
ECS2521_1047	2497693	G (0.93), T (0.07)	[21]
ECS2082_443	2074660	G (0.94), A (0.06)	[21]
ECs0742*	825854	C (0.96), A (0.04)	[21]
ECS2598_1601	2575641	T (0.97), C (0.03)	[21]
ECs5095	5187848	T (0.98), A (0.02)	[21]
ECs5095_115	5188273	G (0.98), A (0.02)	[21]
ECs3957	3961601	T (0.98), A (0.02)	[21]
ECS0975_1973	1066286	C (0.99), G (0.01)	[21]
ECS3141_226	3096645	G (0.99), A (0.01)	[21]
ECS3788_164	3800637	G (0.99), A (0.01)	[21]

*These SNPs are reported to break up cluster 1, according to SNP discovery. Major and minor frequencies are based on 177 isolates.

** One of Manning, *et al*. 32 SNPs

*** One of Leopold, *et al*. cluster one SNPs.

**Fig 2:**
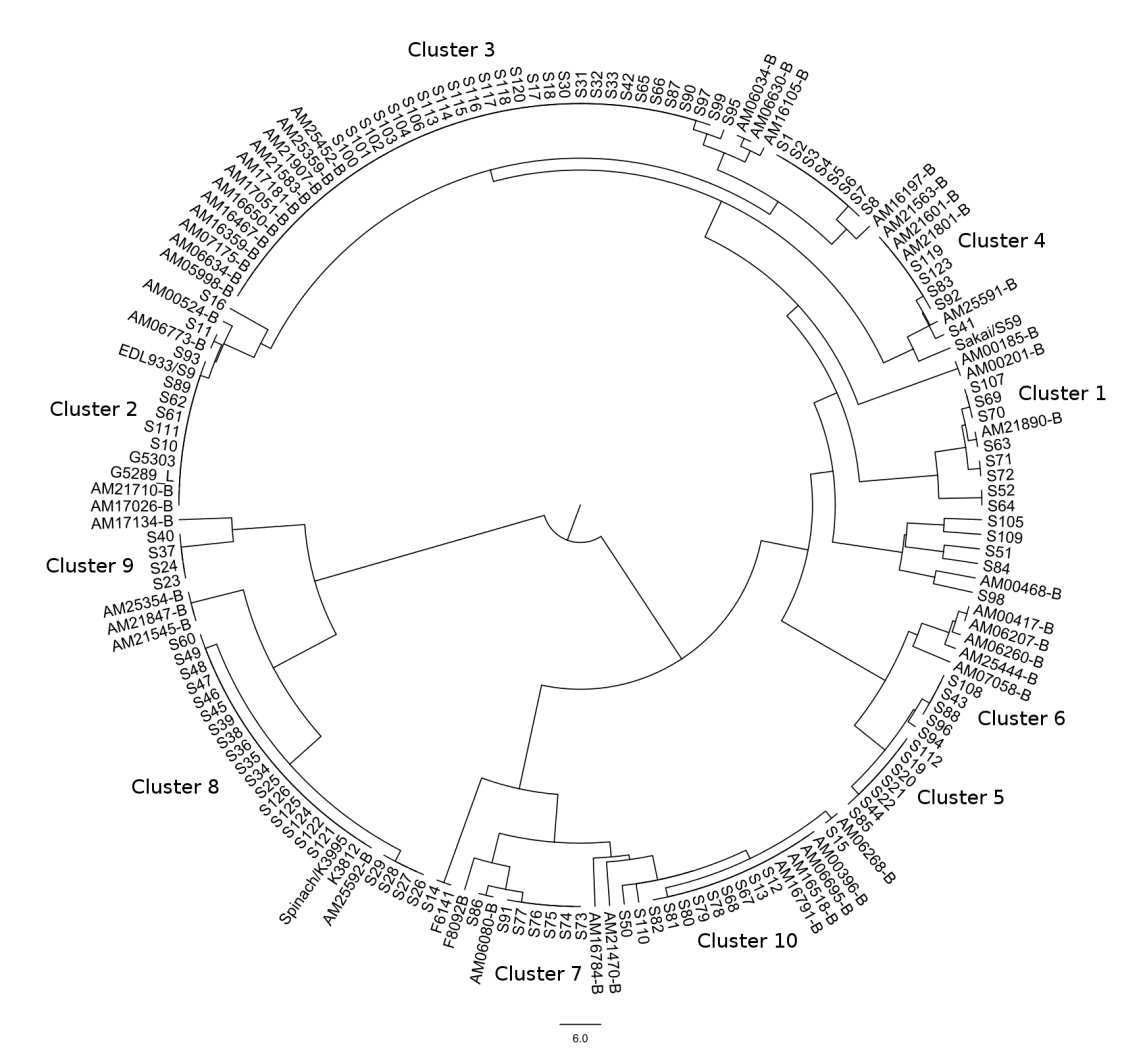
Phylogeny of isolate set using 32 discriminatory SNPs. This is a phylogeny of all isolates used in the study with the exception of a few positive controls shared by other authors using the panel of 32 discriminatory SNPs. The 10 clusters with insufficient epidemiological resolution have been labeled. These clusters are completely different from the three mentioned by Leopold, *et al*. AM series isolates were considered epidemiologically sporadic and should have therefore had additional reticulation in this figure. Some grouping of S-series isolates was expected because some outbreaks were represented by multiple isolates.

We also compared our panel of discriminatory SNPs to the clustering provided by PFGE and MLVA. The SNP panel defined 47 clusters whereas PFGE and MLVA defined 93 and 138 clusters, respectively, when the MLVA test panel, NARMS strains, and positive control isolates were included in analysis (n = 175). Simpson’s D estimates reported a similar trend (Table 3). When the SNP panel was examined from the perspective of MLVA, the SNP panel demonstrated excellent agreement with MLVA (adjusted Wallace value 0.95; Table 3). However, the opposite was not true; when examined from the perspective of the SNP panel, MLVA clusters were not in agreement with SNPs (adjusted Wallace value 0.05). This means that MLVA and the SNP panel were in agreement, but that MLVA had better resolution. A similar result was found when comparing the SNP panel to PFGE, but to a lesser extent (0.44 vs. 0.17). These results imply that our SNP panel had less resolution than MLVA or PFGE. We therefore used the clustering generated by our 32 targets to identify 10 clusters with insufficient epidemiological resolution when applied to the MLVA series isolates. Of these, Clusters 2, 3, 4, 8, and 10 expanded when NARMS strains were included (Fig 2).

**Table 3:** Comparison of PFGE, MLVA and SNP typing.

			Clustering comparison (Wallace)		
	# of Types	Simpson’s Index (D)	PFGE	MLVA	SNP(32)
**PFGE (*Xba*I)**	93	0.9685		0.1072	0.4391
**MLVA**	138	0.9954	0.7534		0.9515
**SNPs**	47	0.9225	0.1701	0.0525	

This table compares the utility and agreement between three subtyping methods. “# of Types” refers to the number of genotypes each method resolved (n = 175). Simpson’s D is a measurement of the amount of diversity found within each method. Finally, the adjusted Wallace comparisons show how much of each method is explained by examining the data from the prospective of another method. MLVA explains almost all the variation seen in the 32 SNP panel.

We looked for additional potentially useful discriminatory SNPs through pooled SNP discovery. We divided 62 of the MLVA series isolates into 10 DNA pools based on the 10 clusters with insufficient epidemiological resolution (S4 Table). This pool verification and discovery process resulted in a raw file with 5495 entries. We reduced the number of discovered SNPs by removing those with less than 99% total coverage of the pool. However, this raw file had duplicate entries for SNPs that occurred in two genes, SNPs that could mistakenly be filtered out by the 99% coverage criteria because they had three alleles, and multiple entries for the same SNP when it occurred in multiple clusters. After accounting for these complications, we were left with 4,040 SNPs. We report summary distributions and detailed cluster and location data in S8–S13 tables. We report the ten SNPs with three or more alleles in S12 Table.

Pool verification suggested that cluster one represented isolates that were incorrectly grouped at some point during our experiments because: 1) We used our 32 SNP panel to sort isolates into cluster 1 and therefore all isolates in this cluster should be homogenous for these alleles, yet the cluster had eight SNPs from our final SNP panel and 2) Cluster one had ~3–8 times the number of internal SNPs (internal SNPs are those that only occurred in that cluster) found in the nine other clusters with insufficient epidemiological resolution (S10 Table), even though many of the other clusters contained more isolates (S4 Table). Therefore, we put information regarding cluster one internal SNPs in our supplementary data file and treated SNPs that occurred in cluster one and one of the other nine clusters as internal SNPs (e.g. a SNP that occurred in cluster one and five was treated as a cluster five internal SNP). Removal of cluster one internal SNPs reduced the novel SNP set from 4,040 to 2,878 targets.

If we discard cluster one SNPs, then pooled SNP discovery verified that our 32 SNP panel captured the discriminatory value of 363 of the initial 391 SNPs, 28 of the 30 SNPs tested on our follow up microarrays, and many additional targets suggested by the literature that we did not examine on initial or follow up microarrays (S5–S7 Table). Comparison to earlier publications suggested that 2,815 of the 2,878 potentially discriminatory SNPs were novel [13, 16, 17, 19, 21, 22]. While cluster one was most likely the result of experimental error, the capability of the 2,878 SNPs to differentiate cluster 1 isolates provides an additional way to discriminate between otherwise similar SNPs (e.g. a SNP that occurs in cluster 1, 4, and 5 may provide a different phylogeny than a SNP that occurs only in cluster 4, and 5).

Pool discovery suggested that five discriminatory SNPs were missed on our initial microarrays and two on our follow up microarrays (again excluding cluster 1 internal SNPs). On our initial arrays, two SNPs (4443619, 4450876) should have reticulated cluster 6 and 10. The remaining three discriminatory SNPs reticulated cluster 2, 3, and 5 respectively (4829856, 1130668, 1444313), as well as cluster 1. On the follow up microarray of the 30 recommended targets, ECs4826 (4891379) should have reticulated cluster 1 and 2 and ECs2775 (2717449) should have reticulated clusters 3, 7, and 9. Fifty targets mentioned by others should have also resolved clusters (excluding internal cluster 1 SNPs) and five of these should have been multi-cluster SNPs; in particular 334265 should have reticulated Clusters 5, 6 and 10 (S6 Table).

## Discussion

Our SNP panel captured almost all the phylogenetic diversity in our 391 SNPs, as well as those recommended by others, when applied to a set of American and Asia isolates that represent the most diverse set of O157:H7 isolates from human outbreaks examined to date. We collapsed hundreds of potential SNPs to 32 informative ones. We used nine clusters with insufficient additional epidemiological resolution to identify 2,878 additional, potentially useful SNPs (S9 and S11 Table). After excluding 16 SNPs we previously reported and 46 reported by others, there were 2,815 novel SNPs for potential future use on discriminatory panels [16, 17, 22].

The phylogeny generated by our 32 SNP candidate panel is generally consistent with those described by MLVA and PFGE, as well as the established epidemiology relationships of tested strains. However, our phylogeny could be biased away from an ideal evolutionary phylogeny because we selected SNPs based on their epidemiological utility. Such bias is unimportant to PulseNet because we are focused on defining epidemiologically meaningful clusters for outbreak investigations, but such bias is important to underscore because it limits our ability to draw evolutionary conclusions.

Regardless, our phylogeny was approximately the same as previous scholarship that did not have a diagnostic bias for branches containing EDL933, Sakai, 93–111, and K3995 (2006 spinach outbreak) [13]. Like others, we found that EDL933 and Sakai represent mutational ‘end points’ with no descendant isolates [16]. The evolutionary insight provided by others may explain why cluster eight was represented by more isolates in our phylogeny than the other clusters. Cluster 8 contains K3995, which may have diverged from other O157 strains 20,000 years ago [21, 22]. Therefore, it might also be expected that this group of isolates would have its own unique SNPs not seen elsewhere in the phylogeny of O157:H7.

Despite our success at identifying a small panel of highly discriminatory SNPs, the panel did not match the subtyping capability of MLVA or PFGE for North American STEC O157:H7 isolates. However, such a panel might be developed based on our panel and a subset of the 2,878 additional SNPs we identified. Among these 2,878 SNPs, 438 SNPs occurred in multiple clusters, excluding those that occur in cluster one and another cluster, as well as SNPs with more than two alleles. Of these, 427 were previously unreported [13, 16, 17, 19, 21, 22]. This suggests the literature has not identified all of the highly discriminatory SNPs available for subtyping *S*TEC O157:H7.

Multi-cluster SNPs may offer the highest discriminatory yield and are thus attractive for future SNP panel development. However, it is likely that the 438 SNPs we identified will collapse to a few discriminatory targets due to linkage disequilibrium. The highest priority targets among the 438 SNPs are the 18 that occurred in six to nine of the clusters (disregarding cluster 1) with insufficient epidemiological resolution. If any of these SNPs provides useful epidemiological discrimination, they would likely eliminate the need to examine many of the other new multi-cluster or internal SNPs we discovered. For example, a multi-cluster SNP might remove the need to examine cluster seven further because it contained only two isolates, even after the inclusion of NARMS isolates. Therefore, these 18 targets should be vetted first for addition to the 32 SNP panel and the resultant phylogeny used to filter through the other multi-cluster SNPs to develop an updated subset of promising multi-cluster SNPs for additional testing.

We have shown that existing SNPs described by our group and the literature are insufficient to create a definitive SNP panel for subtyping North American STEC O157:H7 for epidemiological purposes. However, we recommend a panel of 32 SNPs that eliminate the vast majority of potential SNPs reported for North American STEC O157:H7 and are congruent with other subtyping methods and epidemiological data. Furthermore, we discovered a novel subset of SNPs that may include the additional SNPs required to create a definitive SNP panel for subtyping STEC O157:H7 for epidemiological purposes. Alternatively, a reduced SNP panel may prove insufficient for epidemiological purposes and SNP subtyping may move towards whole genome sequencing as it becomes more economically feasible.

## Supporting Information

S1 Table Initial Data for 903 SNPs. (XLSX)

S2 Table Panel of 32 informative SNPs and Primer/Probes. (XLSX)

S3 Table Strains used in the study. (XLSX)

S4 Table MLVA cluster isolates used in DNA pool discovery. A few isolates were not included in pools because they did not sort into pools until after DNA was already being tested. These samples are noted.
(XLSX)

S5 Table SNPs recommended by others that were not included on follow up microarray. This table describes the SNPs that were considered for use on the follow up microarray and discarded. Note that the “Disrupts tree?” column is based on our pool verification data from the 10 clusters with insufficient epidemiological reticulation as described in the text.
(XLSX)

S6 Table Previously reported SNPs found in clusters other than 1. Leopold 1 and 2 refer to that group's first and second publication regarding E. coli O157:H7.
(XLSX)

S7 Table Previously reported SNPs found only in cluster 1. Leopold 1 and 2 refer to that group's first and second publication regarding *E*. *coli* O157:H7.
(XLSX)

S8 Table SNPs found in multiple clusters. This table is a summary count of SNPs that occurred in two or more of the ten clusters described in the text.
(XLSX)

S9 Table Summarized raw data for multi-cluster SNPs. Each cluster was color coded to make it easier to identify similar SNPs.
(XLSX)

S10 Table Summary of the SNP distribution among clusters and clusters covered by multi-cluster SNPs. The upper summary table summarizes the number of SNPs found in each cluster and breaks them down into categories. “Multi Cluster SNPs” refers to SNPs that were found in multiple clusters. “Special Multi Cluster SNPs” refers to SNPs that had more than two alleles and occurred in multiple clusters. “Internal Cluster SNPs” refers to SNPs that only occurred in a single cluster. “Internal SNPs in Two Genes” refers to internal cluster SNPs that happened to fall within the coding area of two different genes. The column of “Total SNPs” is all of these SNPs added together for each cluster. However, as SNPs occurred in multiple clusters, this column cannot be added together as a count of total SNPs due to redundancy. The last line row called “Total SNPs” is an accurate count of the total number of SNPs in each column class. The lower table is summary of the distribution of multi-cluster SNPs, starting from the maximum of 10 clusters. The first column “Raw Count” represents the raw count of SNPs in each category. “Multi cluster SNPs in two genes” refers to multi-cluster SNPs that occurred in more than one gene. “Clusters including 1” refers to the number of multi-cluster SNPs in each category that included the erroneously identified cluster 1. The final column “Adjusted without 1” refers to an adjusted count of SNPs in each of nine remaining clusters after removing cluster 1 from consideration. “Total ‘useful’ SNPs” refers to the total SNPs that could be explored in the future. “Total 'useful' new SNPs” refers to SNPs that had not been reported previously.
(XLSX)

S11 Table Summarized raw data for internal SNPs. This is raw data for SNPs that only occurred in one of the ten clusters referred to in the text.
(XLSX)

S12 Table SNPs with three possible alleles. This table refers to the few SNPs that either had more than two alleles or occurred in more than gene. Each cluster was color coded to make it easier to identify similar SNPs.
(XLSX)

S13 Table Raw data from cluster SNP discovery. Each cluster was color coded to make it easier to identify similar SNPs.
(XLSX)
